# Immunotoxicity studies of sulfolane following developmental exposure in Hsd:Sprague Dawley SD rats and adult exposure in B6C3F1/N mice

**DOI:** 10.1080/1547691X.2020.1869355

**Published:** 2021-12

**Authors:** AtLee T. D. Watson, Victor J. Johnson, Michael I. Luster, Gary R. Burleson, Dawn M. Fallacara, Barney R. Sparrow, Mark F. Cesta, Michelle C. Cora, Keith R. Shockley, Matt D. Stout, Chad R. Blystone, Dori R. Germolec

**Affiliations:** a Division of the National Toxicology Program, National Institute of Environmental Health Sciences, Research Triangle Park, NC, USA; b Burleson Research Technologies, Inc, Morrisville, NC, USA; c Battelle Memorial Institute, Columbus, OH, USA; d Division of Intramural Research, National Institute of Environmental Health Sciences, Research Triangle Park, NC, USA

**Keywords:** Immunotoxicity, sulfolane, immune system, immunotoxicology, NK cells, innate immunity

## Abstract

Sulfolane is a solvent used in the petrochemical industry and a groundwater contaminant in areas near refineries. The current studies were conducted to assess the impact of oral exposure to sulfolane on the immune system using two models: (1) a perinatal drinking water exposure to 0, 30, 100, 300, or 1000 mg/L from gestation day (GD) 6 until ~ 13 weeks-of-age in Harlan Sprague Dawley rats; and, (2) a 90-day gavage exposure of adult female B6C3F1/N mice to 0, 1, 10, 30, 100, or 300 mg/kg/day. Immune parameters evaluated included measurement of antibody production against sheep red blood cells (SRBC) and keyhole limpet hemocyanin (KLH), *ex vivo* measurements of natural killer (NK) cell activity, cytotoxic T-cell (CTL) activity, and T-cell proliferation, as well as measures of splenic immune cell populations, hematological parameters, and histopathology of immune tissues. A decrease in *ex vivo* NK cell activity was observed in cells from female – but not male – F1 rats following developmental exposure. In adult female mice, splenic NK cell number was lower than the vehicle controls at doses 100 ≥ mg/kg; however, *ex vivo* NK cell activity was not affected by sulfolane treatment. In female mice, a decrease in the number of large unstained cells at doses ≥ 30 mg/kg was observed. In F1 rats, effects on white blood cells (WBC) were limited to a decreasing trend in leukocytes in females; no effects were observed in males. Under the conditions of this study, a no-observed-effect level (NOEL) of 3 mg/kg/day was identified based on reduced NK cell activity in female F1 rats. Overall, these findings suggest that oral exposure to sulfolane in rodents had minimal effects on the immune system.

## Introduction

Sulfolane (2,3,5-tetrahydrothiophene-1,1-dioxide; tetramethylene sulfone) is a high production volume chemical used in liquid-liquid and liquid-vapor extraction of chemicals from petroleum, in fractionalization of wood tars, and as a desulfurization agent in the purification of natural gas. Release of sulfolane into the environment can result in contamination of groundwater and well water in neighboring communities, as evidenced in the city of North Pole, AK where sulfolane has been detected at levels up to 500 parts per billion (ppb) in drinking water (ADEC (Alaska Department of Environmental Conservation) 2012). Therefore, in addition to potential occupational exposure through inhalation and/or dermal routes, residents may potentially receive exposure to sulfolane through ingestion of drinking water and certain foods in these affected communities.

Sulfolane is well absorbed and rapidly eliminated in male and female rats and mice (Waidyanatha et al. 2019, 2020). Acute LD_50_ toxicity values for sulfolane have been observed in the range of 2000 mg/kg in rats and mice (Brown et al. 1966), and other effects on neurobehavior and thermoregulation occur at relatively high doses approaching the LD_50_ (Gordon et al. 1985). A 28-day comparative study demonstrated that rats were more sensitive than mice or guinea pigs when administered sulfolane via gavage, with no-observed-effect levels (NOEL) of 10 and 100 mg/kg reported for rats based on decreased body weights in females and increased kidney and liver weight in males, respectively (Shipkowski et al. 2021).

Reproductive effects have also been observed in rodents following exposure to sulfolane. A guideline reproductive screening study (OECD 421) in Sprague-Dawley rats exposed via gavage to sulfolane (0, 60, 200, or 700 mg/kg/day) during preconception, gestation, and lactation (~63 days) reported increased litter loss, reduced live litter size, and lower pup weights at doses ≥ 200 mg/kg/day (OECD (Organization for Economic Cooperation and Development) 2004, 2016). In mice, a prenatal developmental toxicity study observed increased fetal resorptions and fetal skeletal anomalies at 840 mg/kg (Zhu et al. 1987).

A review of the available toxicity literature suggests that mild-to-moderate decreases in white blood cell (WBC) counts is a relatively consistent finding in animal studies of sulfolane (ATSDR (Agency for Toxic Substances and Disease Registry) 2011). In a 90-day subchronic drinking water study, a no-observed-adverse-effect level (NOAEL) of 25 mg/L (2.9 mg/kg/day) was reported based on decreased WBC, lymphocytes, monocytes, basophils, and large unstained cells (LUC) in female Sprague-Dawley rats. Male rats were unaffected by sulfolane treatment (Huntingdon Life Sciences 2001). The hematological effects in female rats were subsequently used to set a noncancer oral reference dose (RfD) of 0.01 mg/kg/day based on benchmark dose modeling (USEPA (United States Environmental Protection Agency) 2012; Thompson et al. 2013). While severe leukopenia can result in compromised immune function leading to increased susceptibility to infections and cancer, additional data are needed to discern whether mild-to-moderate leukopenia in rodents represents a significant human health effect.

Given the limited toxicity data available for sulfolane, the National Toxicology Program (NTP) conducted studies to evaluate the effects of oral exposure on immune function in two rodent models. Due to continued exposure in communities, which likely included developmental periods, a developmental immunotoxicity study was conducted in Harlan Sprague Dawley rat offspring exposed to 0, 30, 100, 300, or 1000 mg/L in drinking water. In addition, a 13-week repeat dose gavage study was performed in adult female B6C3F1/N mice exposed to 0, 1, 10, 30, 100, or 300 mg/kg/day to provide additional data in a second rodent species as an important aspect of a comprehensive safety assessment. In addition, the adult female mouse study provides a bridge to compare effects following a 13-week exposure with those previously observed following shorter exposure duration in the NTP 28-day gavage studies. Here, we report the effects of sulfolane exposure on a comprehensive suite of immune parameters and functional endpoints to provide necessary toxicity data to inform risk assessment activities for sulfolane.

## Methods

These studies were conducted in compliance with the U.S. Food and Drug Administration Good Laboratory Practices for Nonclinical Laboratory Studies (Title 21 of the Code of Federal Regulations, Part 58).

### Test article

Sulfolane (CAS No. 126–33-0; Lot No. MKBN9784V, > 99% purity) was obtained from Sigma (St. Louis, MO). Sulfolane gavage and drinking water formulations in water (vehicle; tap water from animal facility) were stable for up to 42 days when stored under refrigerated or ambient conditions. All formulations were within ± 10% of target concentrations. Cyclophosphamide (CPS; Lot No MKBS0021V, >99% purity; Sigma) was used as a positive control for immunosuppression. CPS formulations were within 5% of a target concentration of 5 mg/ml.

### Test animals and sulfolane treatment

All animal procedures were approved by the institutional animal care and use committee (IACUC) at the appropriate institution. Female mice (*n* = 8/treatment group) and F1 rats (*n* = 12/sex/treatment group) were randomly assigned to five assay cohorts (Table 1). For F1 rats, no assay cohort contained more than one male or one female from the same litter.

### B6C3F1/N mice

Mouse studies were conducted at Burleson Research Technologies (BRT; Morrisville, NC). Adult female B6C3F1/N mice were obtained from Taconic Biosciences Inc. (Hudson, NY). Female mice were used based on their historical use in NTP immunotoxicity studies to evaluate test article-mediated effects on immune function, and the more robust response in females for both innate and adaptive immunity (Klein and Flanagan 2016). Following an acclimation period of at least 7 days, mice were randomized by body weight ( ± 20% of mean body weight) and group-housed in individually-ventilated cages (up to 5 mice/cage). Municipal tap water (Town of Cary, NC) and NTP-2000 pelleted diet were provided *ad libitum*. All animals were kept in facilities with a 12-h light/dark cycle at 21–24 C and with a 35–65% relative humidity.

Formulations of sulfolane were prepared at concentrations of 0.1, 1, 3, 10, or 30 mg/ml in water to provide dose levels of 0 (vehicle control), 1, 10, 30, 100, or 300 mg/kg/day (based on a dose volume of 10 ml/kg). Exposure levels for female mice were selected based on overt toxicity observed at 800 mg/kg in male and female mice in the NTP 28-day gavage studies (Shipkowski et al., 2021). Female mice (8–9 weeks-of-age at start of dosing) were administered the vehicle or sulfolane daily for 90 days by gavage.

### Harlan Sprague-Dawley rats

The perinatal phase of the rat developmental immunotoxicity studies was initiated at Battelle Memorial Institute (Columbus, OH) as part of a 2-year chronic toxicity study. Time-mated, nulliparous Hsd:Sprague Dawley® SD® (Harlan Sprague Dawley, HSD) rats were obtained from Envigo (Haslett, MI). Following acclimation for up to 15 days, dams were randomized by body weight ( ± 20% of mean body weight) and were housed individually in cages, alone or with their respective litters until weaning on postnatal day (PND) 28. Litters were standardized on PND 4 to eight pups per litter (four/sex, when possible). Post-weaning F1 rats were group housed by sex with up to two males or three females per cage. Male and female F1 rats were shipped to BRT on PND 56–61 for conduct of immune function assays. Additional male and female age-matched HSD rats (*n* = 45/sex) were obtained from Envigo for use as positive control and naïve animals in the immunotoxicity studies. Rats were provided NIH-07 diet from gestation day (GD) 6 to PND 28, and NTP-2000 pelleted feed thereafter *ad libitum*. Vehicle or sulfolane-treated drinking water was provided *ad libitum*. Animal room conditions were maintained on a 12-h light/dark cycle at 21–26 C and with a 35–65% relative humidity.

Time-mated F0 female rats (12–13 weeks-of-age at study initiation) were exposed to vehicle or sulfolane-treated water from GD 6 until PND 28, where GD 0 was defined as the first day of positive evidence of mating. Post-weaning, male and female F1 offspring were exposed to the same dose level as their respective dam from PND 28 until PND 56–61, at which point they were shipped to BRT where exposure continued for 5–6 weeks until study termination on PND 91–95 (for conduct of immune function assays). Formulations of sulfolane were prepared in tap water to provide exposure levels of 0, 30, 100, 300 and 1000 mg/L in drinking water. Exposure levels were selected based on findings of reproductive and developmental toxicity in pregnant rats at doses ≥ 200 mg/kg/day (OECD 2004) and decreased WBC counts and differentials at doses ≥ 400 mg/L in male and female rats (HLS (Huntingdon Life Sciences) 2001). Water bottles were changed twice weekly; water samples were analyzed for both chemical (PACE Analytical, Englewood, OH) and microbial contaminants (Brookside Laboratories, New Brennen, OH).

A positive control group was administered CPS in saline at a dose of 50 mg/kg for mice or 15 mg/kg for rats once per day via intraperitoneal (IP) injection starting on the day of infection or immunization until the day before the scheduled euthanasia, or for the last four days prior to necropsy if no infection or immunization was performed on the cohort.

### General toxicology (Cohort 1)

Food and water consumption were monitored in F1 rats exposed to vehicle or sulfolane in drinking water. Water consumption data was used to calculate sulfolane intake. Daily clinical observations were recorded in all study animals. At necropsy, the liver, spleen, lungs, thymus, kidneys, adrenal glands, bone marrow (femur), gastrointestinal tract with Peyer’s patches (rats only), and mesenteric and popliteal lymph nodes (LN) were collected, fixed in 10% neutral buffered formalin, sectioned at 4–6 mm, and stained with hematoxylin and eosin for histopathological evaluations in the rat study. The lymphoid organs were evaluated using enhanced histopathology (EH) guidelines (Elmore 2006a, 2006b, 2006c, 2006d, 2006e); non-lymphoid organs were evaluated by traditional histopathology. All evaluations were conducted in accordance with the NTP Immunotoxicity Study Pathology Specifications.

### Hematology (Cohort 1)

At their scheduled termination, animals were randomized, rendered unconscious with carbon dioxide (CO_2_), and then blood was collected. Blood (~250 ml) was collected from the retroorbital site and placed into tubes containing K_2_EDTA. Immediately following blood collection and before recovery of consciousness, the animal was returned to the CO_2_ chamber for euthanasia. The blood was analyzed the day of collection on an Advia 120 hematology analyzer using associated V.6.3.2-MS software (Siemens Medical Solutions USA, Inc., Malvern, PA). The following hematologic parameters were assessed: erythrocyte count, hematocrit, hemoglobin concentration, mean cell volume, mean cell hemoglobin, mean cell hemoglobin concentration, platelet count, reticulocyte count, and leukocyte count and differential.

### Assessment of humoral immunity to T-dependent antigens (Cohorts 2 and 3)

Sheep red blood cells (SRBC) in Alsever’s solution (Colorado Serum Company, Denver, CO) were washed three times in phosphate-buffered saline (PBS) and re-suspended in PBS to a final concentration of 3.75 × 10^8^ SRBC/ml for use with the mice or 2 × 10^8^ SRBC/ml for use with the rats. Female mice from Cohort 2 were intravenously immunized via the tail vein with 0.2 ml (7.5 × 10^7^ SRBC) on Study Day (SD) 86. F1 rats from Cohort 2 were intravenously immunized via the tail vein with 0.5 mL (10^8^ SRBC) four days prior to study termination on PND 87–91. Four days after immunization, animals were humanely terminated with CO_2_ and weighed. A maximum amount of blood was collected by cardiac puncture, allowed to clot for 30–60min at room temperature, and serum was then isolated. The serum was stored at −70°C or below until evaluated for anti-SRBC IgM antibodies. The spleen and thymus were removed, and weights recorded.

For the antibody-forming cell (AFC) response to SRBC, spleens from Cohort 2 animals were processed to single cell suspensions in HBSS + HEPES and cell concentration and viability were determined. Spleen cells (1:30 and 1:120 dilution in 100 μl volume) and SRBC (25 μl of 50% suspension in HBSS) were added to 500 μl of molten agar media (at 44 ± 1°C) and mixed with 25 μl of guinea pig complement (1/3 dilution of stock in 1 mL of HBSS with 0.1 mL of 50% SRBC suspension; Cedarlane Laboratories, Burlington, NC) in duplicate tubes. Resulting suspensions were poured onto the center of a petri dish (in duplicate) and covered with glass. The agar was allowed to solidify prior to being placed in an incubator set to maintain 37°C for at least 3h and then AFC plaques were enumerated. The number of plaques were expressed per million spleen cells and per spleen.

Serum samples from Cohort 2 animals immunized with SRBC were also evaluated for anti-SRBC IgM using an ELISA kit (Life Diagnostics, St. Petersburg, FL) according to manufacturer instructions. Following the addition of stop solution, optical density was determined at 450 nm using a Spectramax 340 spectrophotometer and associated Softmax Pro v2.2.1 software (Molecular Devices, Sunnyvale, CA).

Keyhole limpet hemocyanin (KLH; Stellar Biotechnologies, Inc., Los Angeles, CA) whole protein was used as a second T-dependent antigen. Animals from Cohort 3 were immunized 14 days prior to scheduled termination (SD 76 in mice, PND 77–81 in rats) via IP injection of 300 μg KLH/animal. Blood was collected via the saphenous or tail vein, in mice and rats respectively, five days following immunization to examine the impact of sulfolane on the primary IgM antibody response. Terminal blood collection 14 days following immunization with KLH was used to isolate serum for determination of the impact of sulfolane on isotype switching to IgG antibody production. In both cases, isolated sera (stored at 70°C until analyzed) were assayed for anti-KLH IgM or IgG using ELISA kits (Life Diagnostics) as above.ss

### Splenic T-cell proliferation (Cohort 4)

To evaluate T-cell proliferation, 96-well microtiter plates were coated overnight with anti-mouse CD3 antibody (100 μl/well of 1 μg/ml solution of Clone 145–2C11; BD Biosciences) or anti-rat CD3 antibody (100 μl/well of 1 μg/ml solution of Clone G4.18; BD Biosciences) and then washed. Spleen cell suspensions from Cohort 4 animals were separated from red blood cells (RBC) using Ficoll-Paque Plus (GE Healthcare) density gradients. The isolated cells were then re-suspended in complete RPMI 1640 medium (RPMI containing 10% fetal bovine serum (FBS) and 1% penicillin/streptomycin; Gibco ThermoFisher Scientific) at 5 × 10^6^ cells/ml. From this suspension, 100 μl of cells (5 × 10^5^/well) were added to appropriate wells, and the plates incubated at 37 °C (in 5% CO_2_) for up to 72 h. Changes in total cell number, indicative of proliferation, were measured using a fluorescent nucleic acid stain assay (CyQuant Direct Cell Proliferation; ThermoFisher Scientific, Grand Island, NY) according to manufacturer instruct-tions. The fluorescent signal (485 nm excitation/535 nm emission), directly proportional to live cell number and thereby an index of proliferation, was measured using a Spectramax M2e spectrofluorometer and associated Softmax Pro software v5.0 (Molecular Devices).

### Natural killer (NK) cell activity (Cohort 4)

Spleen single cell suspensions from Cohort 4 animals were evaluated for the impact of sulfolane treatment on NK cell killing activity toward tumor target cells (Yang et al. 1994). In brief, spleen effector cells were separated from RBC using Ficoll-Paque Plus density gradient and then separated from adherent cells by passing through nylon wool columns (Polysciences, Inc., Warrington, PA). The spleen effector cell suspensions were diluted in complete RPMI 1640 to achieve effector-to-target ratios of 50:1. 25:1 and 12.5:1. Effector cells (100 μl) were added to triplicate wells in round-bottom microtiter plates that contained 100 μl of target YAC-1 cells (10^4^ cells/well) that had been pre-labeled for 90 min with Chromium-51 (^51^Cr) at a dose of 100 μCi/10^6^ target cells. Spontaneous-release (S) and total ^51^Cr release (T) controls were prepared separately by adding 100 μl of YAC-1 cells to appropriate wells containing 100 μl RPMI 1640 or Triton X-100, respectively. YAC-1 cells (ATCC, Manassas, VA) were cultured and then labeled with ^51^Cr as reported in earlier studies (Burleson et al. 2018).

After loading, the plates were centrifuged at 250 × *g* for five min, then incubated at 37 °C/5% CO_2_ for 4 h. Culture supernatants were harvested and release of ^51^Cr determined using a Cobra II Auto-Gamma counter (Packard, Meriden, CT). Percent-specific ^51^Cr release (NK lysis) was calculated using the formula [(E-S)/(T-S)] × 100, where E is the ^51^Cr release from target cells in the presence of effector cells, S is the spontaneous release of ^51^Cr from target cells alone, and T is the maximum release of ^51^Cr from target cells in the presence of Triton X-100.

### Immunophenotyping (Cohort 4)

Spleen cells from Cohort 4 animals were subjected to ammonium chloride RBC lysis. The resulting mononuclear cells were re-suspended in RPMI with 5% FBS to 2.5 × 10^6^ cells/ml; and 100 μl aliquots containing 2.5 × 10^5^ cells were added to cluster tubes and the cells pelleted. The cells were then re-suspended in 50 μl stain buffer (PBS/2% BSA/0.1% NaN_3_) and incubated for 5–30 min on ice after addition of F_c_ Block solution (BioLegend, San Diego, CA). Following the blocking step, 50 μl of antibody (all BioLegend) cocktails containing antibodies to: (1) CD3, CD161a, CD45, and CD45RA; (2) CD8a, CD3, CD45, and CD4; or (3) for mouse CD11b/CD11c, Ly6G, and NKp46, or rat CD11b/c, CD103, RP-1, and CD161a were added to the appropriate tubes. Antibody staining titers were previously optimized for the experimental conditions and equipment used. Control tubes contained cells only, cells with a single antibody from the list above, or cells with a single isotype control antibody. Cells were then incubated on ice for 20–50 min protected from light. Following incubation, the samples were fixed using 2% paraformaldehyde for at least 30 min, followed by centrifugation and resuspension in fresh stain buffer. The samples were then stored at 2–8 °C, protected from light, until analyzed on an Accuri C6 flow cytometer using CFlow Plus v 1.0.264.15 (BD Biosciences). In all cases, a minimum of 20,000 events/sample was acquired.

Lymphocyte gating was performed on CD45^+^ populations. The following lymphocyte subsets were identified; T-cells (CD3^−^CD45RA), B-cells (CD3^−^CD45RA^+^), NK cells (CD3^−^CD161a^+^), T-helper cells (CD3^+^CD4^+^), and T-cytotoxic cells (CD3^+^CD8^+^). Myeloid cells were gated based on being positive for CD11b with low-to-mid intensity staining for CD11c (mouse) or CD103 (rats). Myeloid populations were differentiated from NK cells based on lack of NKp46 expression, (mouse) or lack of CD161a (rats). Further differentiation was based on expression of Ly6G (mouse) or RP-1 (rat) with positive cells being neutrophils and negative cells differentiated using SSC into monocytes/macrophages with low granularity and eosinophils with high granularity.

### Cytotoxic T-lymphocyte (CTL) assay (Cohort 5)

To stimulate an in vivo cell-mediated immune response, F1 rats in Cohort 5 were infected eight days prior to scheduled termination (PND 83–87) with influenza virus [~2 × 10^5^ plaque forming units (pfu)/rat]; adult female mice were infected with influenza virus (~4 × 10^4^ pfu/mouse) on Day 20 via intranasal instillation (Burleson et al. 2018). The *ex vivo* CTL assay was performed using lung effector cells isolated from the influenza-exposed animals 8 days following infection. Lung effector cells were separated from RBC using Ficoll-Paque Plus density gradient and then separated from adherent cells by passing through nylon wool columns. The resulting single cell suspensions were adjusted using DMEM with 10% FBS and 1% penicillin/streptomycin to achieve effector-to-target ratios of 50:1. 25:1 and 12.5:1. For the assay, 100 μl of the cells were added to 100 μl of target cells (1 × 10^4^ cells/well) that had been both pre-infected with influenza virus and labeled with ^51^Cr as described in Burleson et al. (2018). The U-bottomed plates were then incubated at 37 °C for 6 h before ^51^Cr release was evaluated in each well. The control wells were treated DMEM or Triton X-100 to assess spontaneous and maximum ^51^Cr release, respectively. Percent specific ^51^Cr release (CTL lysis) was calculated via the formula [(E-S)/(T-S)] × 100; where E is the ^51^Cr release from target cells in the presence of effector cells, S is spontaneous release of ^51^Cr from target cells, and T is maximum release of ^51^Cr from target cells. Due to unusually high spontaneous release from the target cells in the mouse CTL assay, CTL-specific killing of the targets could not be determined and is not reported.

### Data collection and statistical analysis

These studies were conducted in compliance with Nonclinical Laboratory Studies Good Laboratory Practice Regulations issued by the U.S. Food and Drug Administration (Title 21 of the Code of Federal Regulations, Part 58). Data were collected into Provantis v9.2.3 (Instem, Philadelphia, PA) and calculation of endpoints was performed within this validated electronic data collection and management system.

Results are presented as mean ± SEM. Jonckheere’s test was used to test for dose-related trends (Jonckheere 1954). Body weight and organ weight data, which typically exhibit a normal distribution, were analyzed using a parametric multiple comparison procedure. If a significant trend was detected at *p* ≤ 0.01, a Williams’ test was used (Williams 1986); if the trend was not significant, a Dunnett’s test was used (Dunnett 1955). Data for other endpoints were analyzed using a non-parametric multiple comparison procedure. If a significant trend was observed Shirley’s test was used (Shirley 1977); if the trend was not significant Dunn’s test was used (Dunn 1964). Positive control group data was compared to the vehicle control group using the Kruskal-Wallis test. Data that were different from control at *p* ≤ 0.05 were considered significant. Extreme values were identified by the outlier test of Dixon and Massey (Dixon and Massey 1957). All flagged outliers were examined by NTP personnel, and implausible values were eliminated from the final analyses.

## Results

Summary findings relevant for evaluating immune toxicity are presented below. All study findings (including individual animal data) are available at the NTP Chemical Effects in Biological Systems (CEBS) database (https://doi.org/10.22427/NTP-DATA-002-03276-0016-0000-7).

### General toxicity

In-life and terminal body weights were not affected by sulfolane treatment in female mice. No clinical indications of toxicity were observed in female mice that were considered related to sulfolane exposure. All female mice that were infected with influenza virus (Cohort 5), regardless of treatment group or control, exhibited clinical signs of piloerection, a commonly occurring symptom following viral challenge.

In rats, water consumption was monitored in F0 dams during gestation and lactation (GD 6 – PND 28) and in F1 offspring from PND 35 to approximately PND 91 to determine sulfolane intake (Table 2). No clinical signs of toxicity were observed in sulfolane-exposed F0 dams, and there were no apparent effects on gestational and lactational weight gain, litter parameters, or pup weights on PND 0. At weaning on PND 28, male and female F1 offspring weighed 7–8% less than controls in the 1000 mg/L group (data not shown). Water consumption in male and female F1 rats at all test doses was not statistically different from the vehicle controls for the duration of the study. When normalized to body weight, sulfolane exposure in female F1 rats was up to 63% higher than that of male F1 rats on a mg per kg-day basis. Feed consumption was 6–7% lower than controls in male and female rats exposed to 1000 mg/L during the post-weaning period. In female F1 rats, there was a significant trend in lower body weights with increasing dose during the post-weaning period, with 1000 mg/L females weighing 7–10% less than the vehicle control group from PND 35 until study termination on PND 91. Male F1 rats in the 100 mg/L group weighed ~5% less when compared to the vehicle control group for the during the post-weaning period. No clinical indications of toxicity that were considered related to sulfolane exposure were noted in F1 rats.

### Organ weights and pathology

In female mice from Cohort 1 there was a positive trend in liver weight with a significant increase (+16%) in the 300 mg/kg treatment group compared to that of the vehicle control group. No other notable changes in organ weights were observed in female mice treated with sulfolane; female mice treated with CPS demonstrated marked decreases in spleen and thymus weights.

Exposure to sulfolane in female F1 rats resulted in significant increases in absolute and relative thymus weights (+18%) in the 300 mg/L group, and a negative trend with a significant decrease in absolute kidney weight (−9%) in the 1000 mg/L group compared to the vehicle control group (Table 3). In male F1 rats, absolute and relative spleen weights demonstrated a positive trend with increasing sulfolane exposure, with a pairwise significant increase of 11% when compared to controls in relative spleen weights in the 1000 mg/L group. Spleen and thymus weights were significantly decreased in CPS-treated male and female F1 rats (Table 3).

There were no gross or microscopic changes identified in lymphoid tissues (spleen, thymus, lymph nodes, BALT, bone marrow) or in non-lymphoid tissues examined (liver, lung, right kidney, right adrenal gland) in female mice or F1 rats that were attributed to sulfolane exposure. There were a number of gross and histopathological findings identified but these were considered sporadic or background findings based on their low incidence, minimal severity, and/or similar incidence between control and treated groups.

### Hematology

Exposure to sulfolane in female mice and F1 rats resulted in changes in a few hematological parameters. A negative decreasing trend in LUC count was observed in female mice with significant pairwise decreases observed at doses ≥ 30 mg/kg (Table 4). In female F1 rats, a negative trend was observed in leukocyte counts and a positive trend was observed in reticulocyte counts; however, no pairwise differences were observed in individual treatment groups when compared to the vehicle controls. No treatment-related differences were observed in the leukogram of the male F1 rats. There were no other changes in the hematology data from mice or F1 rats that were considered related to sulfolane exposure. CPS-treated female mice and F1 rats showed characteristic decreases in most hematological parameters including reticulocyte and erythrocyte counts, as well as the leukocyte count and differential.

### Ex vivo *T-cell proliferation*

The ability of monoclonal anti-CD3 antibodies to induce T cell proliferation was used as a measure of T-cell function. A dose-related trend in lower CD3-mediated *ex vivo* proliferation of T-cells was noted with the female mice. There were no exposure-related effects on T-cell proliferation in spleen cells isolated from either female or male F1 rats exposed to sulfolane. CPS treatment caused a significant decrease in *ex vivo* T-cell proliferation in spleen cells isolated from the female mice and male F1 rats but not from female F1 rats.

### Ex vivo *natural killer (NK) cell activity*

**Figure 1.**
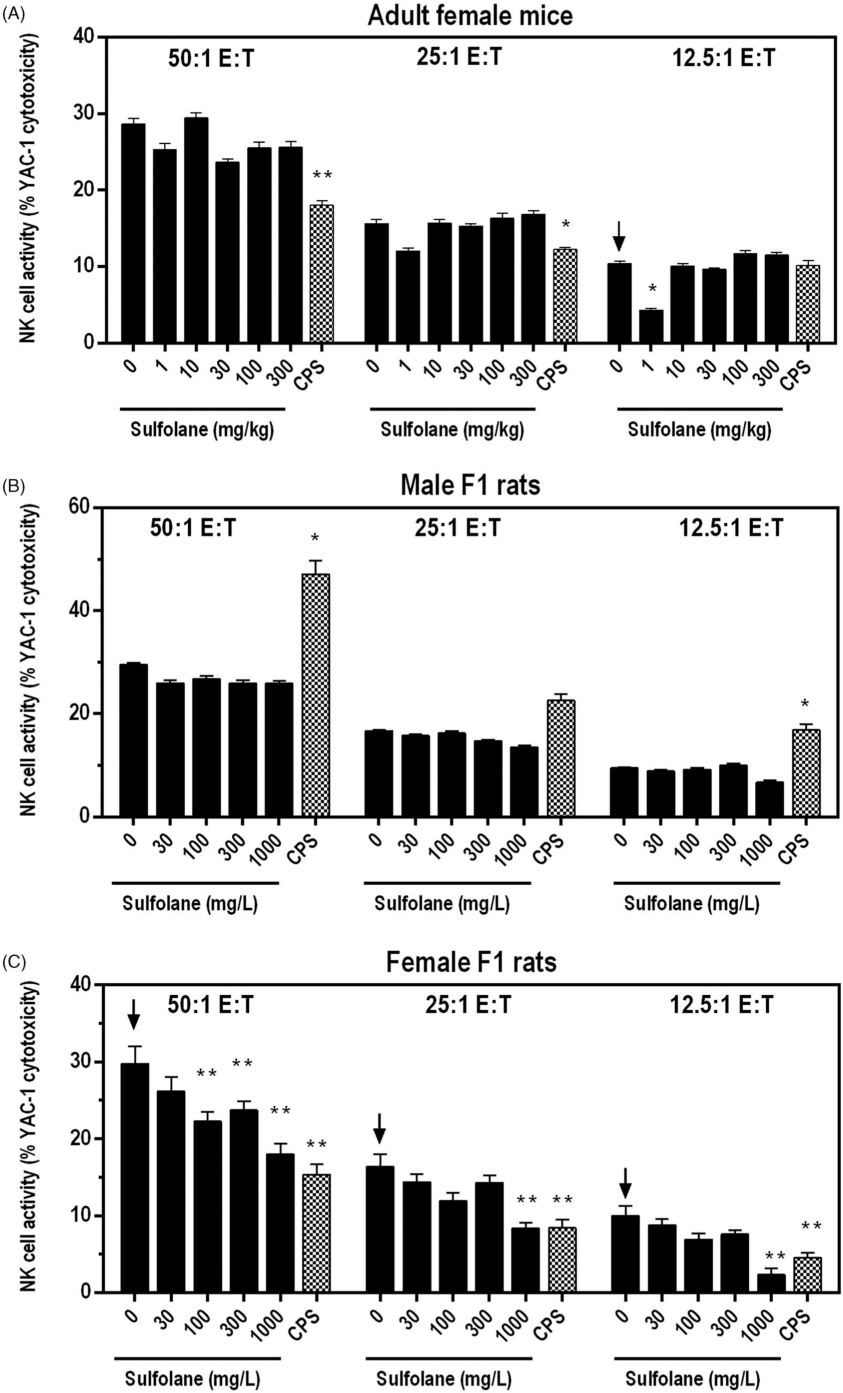
NK cell activity following exposure of adult female B6C3F1/N mice and F1 rats to sulfolane. Cyclophosphamide (CPS) administered via IP intraperitoneal injection at 15 mg/kg (rat) or 50 mg/kg (mice). Arrows indicate significant decreasing trend with increasing sulfolane exposure (*p*< 0.01 for mice and *p*< 0.05 for rats). Result is significantly different from vehicle control group (*p< 0.05; **p< 0.01). E:T: effector:target ratio.

Natural killer (NK) cell activity was evaluated by examining the ability of splenic NK cells to lyse YAC-1 tumor target cells. NK activity was significantly decreased at the 12.5:1 E:T ratio among cells isolated from female mice treated with 1 mg/kg sulfolane relative to the vehicle control group. *Ex vivo* NK activity in all other sulfolane treatment groups in female mice was similar to the vehicle control group (Figure 1(A)).

Sulfolane treatment had no significant effects on *ex vivo* NK cell activity in cells isolated from male F1 rats (Figure 1(B)). In contrast, NK cell activity decreased in a dose-dependent manner in cells from the sulfolane-treated female F1 rats. NK cell activity was significantly decreased up to 47% at exposure levels of ≥ 100 mg/L at the 50:1 E:T ratio and was reduced by 49% at 1000 mg/L at the 25:1 E:T ratio. Cells isolated from female F1 rats treated with 1000 mg/L of sulfolane demonstrated reduction of NK cell activity of 40–76% at all E:T ratios tested when compared to cells from the vehicle controls (Figure 1(C)). NK cell activity was significantly decreased in cells from CPS-treated female mice (50:1 E:T and 25:1 E:T ratios) and female rats (all E:T ratios) and was increased in male rats.

### Immunophenotyping

**Figure 2.**
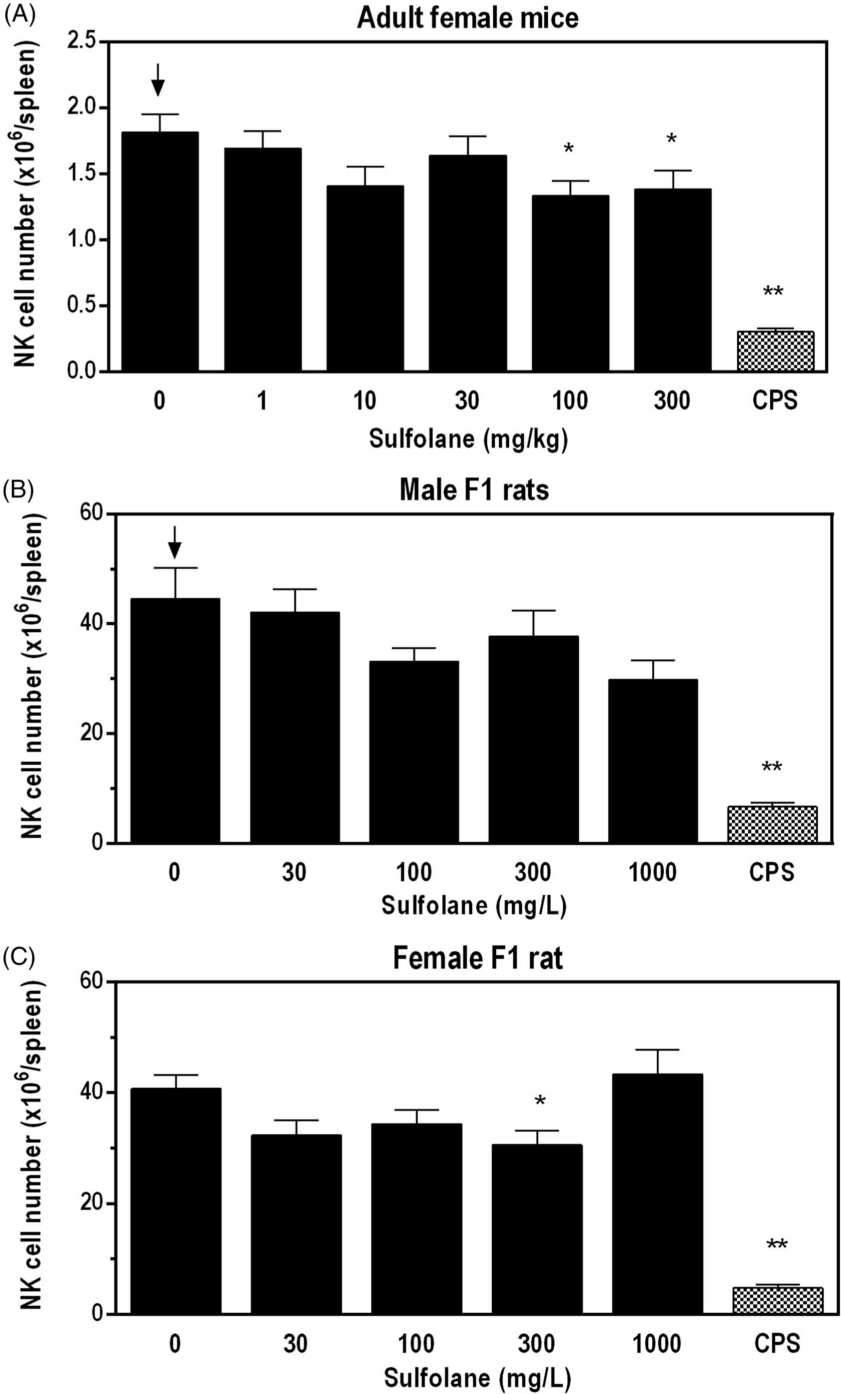
Splenic NK cell numbers following exposure of adult female B6C3F1/N mice and F1 HSD rats to sulfolane. Cyclophosphamide (CPS) administered via IP injection at 15 mg/kg (rat) or 50 mg/kg (mice). Arrows indicate a significant decreasing trend with increasing sulfolane exposure (*p*< 0.05). Result is significantly different from vehicle control group (*p< 0.05; **p< 0.01).

Spleen cell enumeration was performed to determine absolute and relative numbers of immune cell populations for female mice and F1 rats (Tables 5 and 6, respectively). In female mice, lower numbers of NK cells (24–27%) and the relative percentage of NK cells compared to vehicle controls were observed at doses ≥ 100 mg/kg (Figure 2(A)). In addition, there was a small, but statistically significant decrease in the percentage of total lymphocytes (~2% lower than controls) at doses ≥ 30 mg/kg in female mice.

In male F1 rats, there were negative trends in total spleen cells, NK cells, and neutrophils. Additional changes in male F1 rats included lower numbers of monocytes/macrophages (−31%) and neutrophils (−32%) with exposure to 1000 mg/L when compared to the vehicle controls. Although not statistically significant, NK cell numbers were 33% lower than the vehicle controls in male F1 rats at the 1000 mg/L exposure level (Figure 2(B)). In female F1 rats, there was a significant decrease in the numbers of NK cells (−25%) (Figure 2(C)) and decrease in eosinophils (−26%) at 300 mg/L, and a significant increase in the relative percentage of T-cells at 100 mg/L when compared to vehicle controls. In both male and female F1 rats, there was a positive trend for an increase in the percent of B cells.

Immunophenotype profiles observed in the CPS treated female mice and male and female rats demonstrated significant decreases in nearly all cell populations when expressed as absolute cell numbers (Tables 5 and 6).

### Ex vivo *cytotoxic T-lymphocyte (CTL) activity*

*Ex vivo* CTL responses were unaffected following sulfolane treatment of female F1 rats. With male F1 rats, sporadic changes in *ex vivo* CTL activity were observed at select exposure levels and E:T ratios. A modest though not statistically significant decrease was observed in cells from the male F1 rats exposed to 1000 mg/L sulfolane when the cells were assayed at the 12.5:1 and 25:1 E:T ratios. A trend toward lower CTL activity was also observed at the 50:1 E:T ratio; however, changes in individual exposure groups were minimal (≤ 24%) and did not significantly differ (*p* < 0.05) compared to cells from the vehicle control group. The CTL response in cells from the CPS-treated rats was significantly decreased at all E:T cell ratios. In female mice, CTL-specific cytotoxicity could not be resolved due to the high spontaneous background release of ^51^Cr from the target cells (in the absence of lung effector cells).

## Discussion

Given the potential for human exposure in communities near oil and natural gas refineries where sulfolane contamination has occurred, and previous studies that suggest the immune system may be a target, the potential for sulfolane to modulate immune function in adult female mice and developing male and female rat offspring was evaluated. Overall, sulfolane exposure resulted in reduced NK cell activity in spleen cells obtained from female F1 rats and changes in splenic immune cell populations in female mice and F1 rats; several minimal changes in the leukograms of female mice and male and female F1 rats were also observed.

Of the functional endpoints evaluated, sulfolane treatment had the most dramatic effect on NK cell parameters. Across species and sexes, slight reductions (24–33%) in NK cell numbers at select dose and exposure levels were observed. In female F1 rats, *ex vivo* NK cell lytic function against YAC-1 tumor cells was significantly reduced in an exposure-dependent manner; however, despite similar reductions in NK cell number, significant reductions in NK cell activity with cells from adult female mice or male F1 rats after exposure to sulfolane were not observed. This observed difference between male and female rats could be attributed to the higher exposure levels in female F1 rats compared to their male counterparts.

An absence of suppressed NK cell activity in female mice and male F1 rats is not necessarily surprising given the existence of multiple intra-/extracellular targets whose modulation could impair NK cell function at exposure concentrations that do not induce NK cell apoptosis/necrosis. For example, *ex vivo* NK cell activity in mice is suppressed following exposure to the carbamate pesticide metam (Pruett et al. 1992) as well as to the herbicide propanil and its metabolite 3,4 dichloroaniline (Barnett 1992). Mechanistic studies in human NK cell lines demonstrate that the carbamates ziram and thiram cause lower intracellular perforin and granzyme levels, which mediate NK cell cytotoxicity against tumor cells and viral pathogens (Li et al. 2012, 2015). Similar *in vitro* studies with pentachlorophenol have shown reduced human NK cell lytic function due to reduced ATP levels and/or NK cell binding capacity (Hurd et al. 2012), or in the case of atrazine, through inhibition of cytolytic granule release (Rowe et al. 2007). While elucidation of these potential modes of action is beyond the scope of the present study, it is plausible that one or more of these factors could be responsible for observed reduction in NK lytic function in sulfolane-treated female F1 rats.

Given the role of NK cells as innate effectors against viral infections and tumor cells, it is important to understand what degree of NK cell suppression results in increased susceptibility to infection or tumorigenesis. In the present study, sulfolane exposure in female F1 rats suppressed *ex vivo* NK lysis of YAC-1 tumor cells in a dose-dependent manner with 40–76% lower activity observed at the highest exposure level of 1000 mg/L across the E:T ratios tested. For context, a prior study in NK cell-depleted mice challenged with B16F10 melanoma cells found that tumor burden was increased when NK cell activity was reduced by more than 80%. However, recognizing that the immune response reflects both the strength of the host response and the nature of the challenge, the same study found tumor burden was increased when NK cells activity was suppressed by only ~50% when the number of B16F10 cells was increased (Wilson et al. 2001). Importantly, a similar magnitude of reduced *ex vivo* NK cell activity in sulfolane-exposed F1 females was observed; however, the ability of these animals to respond to tumorigenic challenges in vivo remains unknown.

The importance of NK cell function and its modulation by genetic factors and chemical and non-chemical stressors is well established. In humans, NK cell deficiency (NKD) disorder, a primary immunodeficiency disease in patients with NK cell-specific abnormalities, is characterized by reduced NK cell numbers, subsets and/or function (as reviewed by Mace and Orange 2019). The clinical consequences of NKD include fatal viral infections and increased susceptibility to latent viral infections such as cytomegalovirus and herpes viral infections. In addition, there is some epidemiological evidence to suggest an association between exposure to certain pesticides, some of which have been shown to alter NK cell function in mice (mentioned above), and an increased risk of developing non-Hodgkin’s lymphoma (McDuffie et al. 2001; Fritschi et al. 2005).

Further, numerous studies report impaired immune function in individuals experiencing prolonged psychological stress (as reviewed by Webster Marketon and Glaser 2008). Lower NK cell activity has been reported among medical students (Kiecolt-Glaser et al. 1986), long-term patient caregivers (Irwin et al. 1991), and those experiencing depression (Irwin et al. 1992) and marital stress (Kiecolt-Glaser et al. 1993). Stress-related immunosuppression has been seen in mice where stress induced by physical restraint resulted in lower NK cell and CTL activity and led to increased viral titers in mice challenged with herpes simplex type 1 virus (Bonneau et al. 1991). While no human studies examining immune responses in individuals exposed to sulfolane have been published, it is important to recognize the multifactorial nature of the human immune system and its potential modulation by various chemical and non-chemical stimuli. As a result, these stimuli could exacerbate individual sensitivities in stressed or immunocompromised individuals that may not be detected in standard immune assays conducted in laboratory animals.

Additional effects were observed in select hematological parameters. Decreased numbers of large unstained cells (LUC) were observed in female mice; however, similar trends were not seen with other leukocyte populations. LUCs are counted by some automated hematology analyzers. These cells cannot be identified by the instrument into one of the five major cell types, but are generally thought to be monocytes, lymphocytes or some immature cells; large increases in LUC would warrant evaluation of a peripheral blood smear. Decreases of LUC without any other changes in the leukogram have uncertain relevance. While there were no notable hematological effects observed with the male F1 rats, there was a negative trend (no pairwise differences) in leukocyte counts of female F1 rats; this was largely driven by the changes observed with the highest dose. This is somewhat consistent with the negative trend in leukocytes observed with female and male rats following a 28-day repeat dose gavage exposure (Shipkowski et al. 2021). In addition, the female-specific effects on leukocytes and lymphocytes in the present study, although lower in magnitude, are consistent with those observed in a subchronic (90-day) drinking water study (HLS Huntingdon Life Sciences 2001) in that female and not male rats were affected. Here, decreases of 20 and 22% in lymphocytes and leukocytes, respectively, were observed in the 1000 mg/L (~128 mg/kg/day) female F1 rats compared to respective decreases of 47 and 43% in female rats in the Huntingdon Life Sciences study receiving 1600 mg/L in drinking water (~191 mg/kg/day). The reason for the quantitative differences is unclear but may be due to the difference in timing (e.g. developmental vs. adult exposure) or magnitude of exposure.

It is also worth noting the different oral exposure routes in this study, especially in relation to the known toxicokinetic and absorption, distribution, metabolism, and elimination (ADME) properties of sulfolane. Sulfolane is rapidly absorbed and cleared in male and female B6C3F1/N mice and HSD rats, with no apparent sex differences in ADME or toxicokinetic parameters following a single gavage exposure to 10, 30, or 100 mg/kg) (Waidyanatha et al. 2020). At the same dose levels, B6C3F1/N mice reach a similar C_max_ but have shorter plasma elimination half-lives (< 1.3 h) compared to HSD rats (< 6.3 h) when administered sulfolane via gavage, with saturation of clearance pathways beginning at 30 mg/kg (Waidyanatha et al. 2019).

In the present study, female mice received sulfolane as a bolus once-daily gavage; in comparison, drinking water exposure to F1 rats resulted in a more gradual ingestion of sulfolane throughout the 24-h period. Moreover, the highest exposure level in adult female mice (300 mg/kg/day) was two- to four-fold higher than that of the male and female F1 rats (78 and 128 mg/kg/day, respectively). The minimal to no immune-related effects of sulfolane in adult female mice was likely due to faster clearance of sulfolane in the mice as compared to in the rats. The reduction of NK cell activity in female F1 rats at exposure levels ≥ 100 mg/L (≥ 10.2 mg/kg/day) may reflect an overall greater sensitivity to sulfolane in rats compared with mice, as previously shown by Shipkowski et al. (2021). However, the precise mechanisms underlying the female-specific reduction in NK cell activity in rats remains uncertain.

## Conclusions

As part of a broader toxicological evaluation of sulfolane by the NTP, the present studies provide a comprehensive assessment of sulfolane exposure on immune function in adult female mice and developmentally-exposed F1 rats. Contamination of drinking water with sulfolane has been confirmed in select communities neighboring oil and gas refineries, and thus, it is critical to understand the potential impacts of sulfolane under human relevant exposure scenarios (e.g. drinking water). Under the conditions of this study, female F1 rats demonstrated the greatest sensitivity to sulfolane exposure with a no-observed-effect level (NOEL) of 3mg/kg/day based on the exposure-dependent reduction of NK cell activity. These effects occurred at exposure levels two orders of magnitude higher than current oral RfD of 0.01mg/kg/day. In addition, select hematological and splenic lymphocyte parameters were altered; however, the majority of changes in cell populations were either sporadic or low in magnitude, and were not consistently altered across species, gender, and timing of exposure. Overall, these findings suggest that oral exposure to sulfolane in rodents had minimal effects on the immune system.

## Figures and Tables

**Table 1.** Study cohorts and endpoints assessed in sulfolane-treated adult female B6C3F1/N mice (*n* = 8/treatment group) and F1 Harlan Sprague Dawley rats (*n* = 12/sex/treatment group).

Cohort	Endpoints examined
1	Gross pathology
	Enhanced immunopathology
	Hematology (Complete blood count with differential and reticulocyte counts)
2	Antibody response to SRBC
3	Antibody response to KLH
4	T-cell proliferation
	NK cell activity
	Immunophenotyping of the spleen
5	CTL response to influenza infection

CTL: cytotoxic T-lymphocyte; KLH: keyhole limpet hemocyanin; NK: natural killer; SRBC: sheep red blood cell.

**Table 2.** Mean water consumption and sulfolane intake during the perinatal and post-weaning period in HSD rats (Cohort 1) exposed to sulfolane in drinking water.

	Generation/sex	Sulfolane in drinking water (mg/L)				
		0	30	100	300	1000
Gestation (GD 6–21)						
Water consumption^a^	F_0_/F	0.0 ± 0.0	121.4 ± 2.7	121.9 ± 2.0	120.9 ± 2.0	127.1 ± 2.9
Sulfolane intake^b^		0.0 ± 0.0	3.6 ± 0.1	12.2 ± 0.2	36.3 ± 0.6	127.1 ± 2.9
Lactation (PND 1–28^c^)						
Water consumption		0.0 ± 0.0	254.6 ± 5.9	266.7 ± 5.9	267.7 ± 6.5	270.0 ± 6.8
Sulfolane intake		0.0 ± 0.0	8.0 ± 0.2	26.8 ± 0.6	80.8 ± 1.1	270.0 ± 6.8

Postweaning (PND 35–91)						
Water consumption	F_1_/M	86.3 ± 1.8	85.8 ± 3.8	95.3 ± 2.4	84.0 ± 2.2	78.4 ± 2.2
	F_1_/F	104.3 ± 3.5	109.6 ± 2.4	101.7 ± 1.9	110.3 ± 3.8	127.7 ± 2.3
Sulfolane intake	F_1_/M	0.0 ± 0.0	2.6 ± 0.1	9.5 ± 0.2	25.2 ± 0.7	78.4 ± 2.3
	F_1_/F	0.0 ± 0.0	3.3 ± 0.1	10.2 ± 0.2	33.1 ± 1.1	127.7 ± 2.3

Mean ± SE. *N* = 30–38/treatment group (F_0_ dams); *N* = 12/treatment group (F_1_ offspring).

a Units are in g water/kg BW/day.

b Units are in mg sulfolane/kg BW/day.

c Reported for F0 females but includes consumption by F1 offspring beginning on or around PND 13 until weaning on PND 28.

**Table 3.** Organ weights in male and female F1 rats exposed developmentally to sulfolane.

	Sulfolane (mg/L)					
	0	30	100	300	1000	CPS (*n* = 8) 15 mg/kg
F1 MALES						
Terminal BW	449.0 ± 7.4	453.0 ± 7.8	425.7 ± 3.3	443.1 ± 10.0	438.7 ± 5.5	**364.8 ± 5.9** **
Spleen	0.75 ± 0.02^#^	0.76 ± 0.03	0.73 ± 0.02	0.79 ± 0.03	0.82 ± 0.03	**0.45 ± 0.01** **
Spleen:BW ratio	1.67 ± 0.04^##^	1.68 ± 0.05	1.70 ± 0.04	1.79 ± 0.04	**1.86 ± 0.06** **	**1.24 ± 0.05** **
Thymus	0.40 ± 0.02	0.41 ± 0.02	0.37 ± 0.02	0.41 ± 0.03	0.38 ± 0.02	**0.18 ± 0.01** **
Thymus: BW ratio	0.88 ± 0.03	0.90 ± 0.04	0.88 ± 0.05	0.92 ± 0.05	0.86 ± 0.04	**0.49 ± 0.03** **
Adrenal	0.024 ± 0.001^#^	0.022 ± 0.001	0.022 ± 0.001	0.021 ± 0.001	0.021 ± 0.001	**0.021 ± 0.001** *
Adrenal:BW ratio	0.05 ± 0.00	0.05 ± 0.00	0.05 ± 0.00	0.05 ± 0.00	0.05 ± 0.00	0.06 ± 0.00
Liver	16.4 ± 0.5	16.6 ± 0.4	15.6 ± 0.3	15.9 ± 0.6	16.7 ± 0.4	**12.5 ± 0.4** **
Liver:BW ratio	36.4 ± 0.7	36.7 ± 0.4	36.4 ± 0.6	35.7 ± 0.6	37.9 ± 0.7	34.1 ± 0.9
Kidney	1.53 ± 0.03	1.53 ± 0.03	1.45 ± 0.03	1.48 ± 0.04	1.55 ± 0.03	**1.19 ± 0.05** **
Kidney:BW ratio	3.42 ± 0.08	3.37 ± 0.05	3.41 ± 0.08	3.35 ± 0.06	3.54 ± 0.06	3.25 ± 0.10
F1 FEMALES						
Terminal BW	265.3 ± 4.5^#^	264.8 ± 6.2	274.8 ± 5.4	263.3 ± 7.3	246.8 ± 2.5	**216.9 ± 4.0** **
Spleen	0.56 ± 0.02	0.58 ± 0.02	0.581 ± 0.02	0.58 ± 0.03	0.58 ± 0.02	**0.37 ± 0.01** **
Spleen:BW ratio	2.11 ± 0.06	2.20 ± 0.05	2.11 ± 0.05	2.20 ± 0.09	2.36 ± 0.08	**1.68 ± 0.04** **
Thymus	0.27 ± 0.01	0.28 ± 0.01	0.29 ± 0.01	**0.31 ± 0.02** *	0.25 ± 0.01	**0.15 ± 0.02** **
Thymus: BW ratio	1.01 ± 0.03	1.07 ± 0.03	1.06 ± 0.05	**1.19 ± 0.06** *	1.02 ± 0.07	**0.68 ± 0.07** **
Adrenal	0.032 ± 0.001	0.029 ± 0.001	0.033 ± 0.001	0.032 ± 0.001	0.030 ± 0.001	0.032 ± 0.002
Adrenal:BW ratio	0.12 ± 0.00	0.11 ± 0.00	0.12 ± 0.00	0.12 ± 0.01	0.12 ± 0.00	**0.15 ± 0.01** *
Liver	9.34 ± 0.30	9.32 ± 0.35	9.52 ± 0.22	9.03 ± 0.37	9.15 ± 0.26	**7.09 ± 0.16** **
Liver:BW ratio	35.3 ± 1.1	35.2 ± 1.0	34.3 ± 0.5	34.2 ± 0.7	37.1 ± 0.9	32.7 ± 0.53
Kidney	0.92 ± 0.02^#^	0.90 ± 0.02	0.94 ± 0.03	0.87 ± 0.02	**0.84 ± 0.02** *	**0.78 ± 0.02** **
Kidney:BW ratio	3.46 ± 0.07	3.41 ± 0.06	3.43 ± 0.08	3.30 ± 0.07	3.40 ± 0.07	3.59 ± 0.06

Values represent mean ± SEM (*n*= 11–12/group, unless noted otherwise).

Absolute weights in g; organ:BW ratios in mg/g BW.

BW: bodyweight; CPS: cyclophosphamide.

# Significant decreasing trend *p* < 0.05;

## *p* < 0.01.

Bold values indicate significant pairwise differences when compared to the vehicle control group

* *p* < 0.05;

** *p* < 0.01.

**Table 4.** Select hematological parameters following daily gavage exposure to sulfolane in female mice.

	Sulfolane (mg/kg)						
	0	1	10	30	100	300	CPS
Animals (*N*)	7	5	6	7	6	7	7
Leukocyte Count	5.86 ± 0.81	7.57 ± 0.84	6.45 ± 0.37	4.97 ± 0.56	5.50 ± 0.52	6.54 ± 1.23	**1.60 ± 0.14** **
Lymphocyte Count	4.84 ± 0.66	6.31 ± 0.66	5.11 ± 0.40	4.11 ± 0.50	4.49 ± 0.45	5.21 ± 1.01	**1.38 ± 0.11** **
Neutrophil Count	0.71 ± 0.11	0.72 ± 0.12^1^	0.80 ± 0.16	0.53 ± 0.05	0.67 ± 0.05	0.87 ± 0.14	**0.14 ± 0.03** **
Monocyte Count	0.13 ± 0.02	0.15 ± 0.01	0.11 ± 0.02	0.11 ± 0.01^1^	0.13 ± 0.02	0.13 ± 0.02	**0.02 ± 0.01** **
Eosinophil Count	0.10 ± 0.02	**0.26 ± 0.04** *	0.34 ± 0.11	0.13 ± 0.03	0.15 ± 0.03	0.19 ± 0.07	**0.04 ± 0.02** **
Basophil Count	0.02 ± 0.00	0.05 ± 0.02	0.03 ± 0.01	0.01 ± 0.00	0.02 ± 0.01	0.02 ± 0.01^1^	**0.003 ± 0.00** **
LUC Count	0.07 ± 0.02^##^	0.07 ± 0.01	0.06 ± 0.01	**0.03 ± 0.01** **	**0.04 ± 0.00** *	**0.05 ± 0.01** *	**0.03 ± 0.01** *

Values represent means ± SEM.

Counts in 10^3^ cells/ll.

CPS: cyclophosphamide (50 mg/kg); LUC: large unstained cell.

## Significant dose-related trend *p* < 0.01.

Bold values indicate significant pairwise differences when compared to the vehicle control group

* *p* < 0.05;

** *p* < 0.01.

1 *N* = 6 due to removal of outliers.

**Table 5.** Splenic immunophenotyping following gavage exposure to sulfolane in female B6C3F1/N mice.

	Sulfolane (mg/kg)						
	0	1	10	30	100	300	CPS
Total spleen cells	73.7 ± 6.7	67.7 ± 3.8	66.3 ± 5.7	67.0 ± 4.8	59.5 ± 2.4	69.3 ± 6.3	**16.3 ± 1.5** **
Total lymphocytes	73.1 ± 6.6	66.9 ± 3.8	65.3 ± 5.6	65.7 ± 4.8	58.2 ± 2.3	68.0 ± 6.1	**15.9 ± 1.5** **
Total lymphocytes (%)	99.3 ± 0.1^##^	98.9 ± 0.3	98.6 ± 0.3	**98.0 ± 0.4** **	**97.8 ± 0.5** *	**98.2 ± 0.2** **	**97.3 ± 0.6** **
Total T cells	23.7 ± 2.3	19.9 ± 1.2	19.2 ± 2.1	20.0 ± 1.9	18.4 ± 0.8	21.1 ± 2.0	**7.69 ± 0.72** **
Total T cells (%)	32.3 ± 0.8	29.8 ± 0.4	29.3 ± 1.3	30.3 ± 1.0	31.3 ± 0.5	31.2 ± 1.2	**48.4 ± 0.8** **
CD4^+^ T cells	14.2 ± 1.5	11.7 ± 0.7	11.4 ± 1.1	12.2 ± 1.1	10.6 ± 0.5	11.9 ± 1.1	**3.87 ± 0.42** **
CD4^+^ T cells (%)	59.6 ± 1.0	58.6 ± 0.4	59.9 ± 1.6	61.1 ± 1.7	57.8 ± 0.6	56.6 ± 1.0	**50.0 ± 1.0** **
CD8^+^ T cells	8.56 ± 0.87	7.50 ± 0.56	7.11 ± 0.86	7.30 ± 0.58	6.63 ± 0.33	7.33 ± 0.79	**2.90 ± 0.25** **
CD8^+^ T cells (%)	36.0 ± 0.6	37.4 ± 0.6	36.7 ± 0.9	36.9 ± 1.1	35.6 ± 0.6	34.5 ± 0.9	**38.0 ± 0.7** *
B cells	43.5 ± 3.8	41.6 ± 2.2	40.8 ± 3.4	39.7 ± 2.9	35.4 ± 1.4	42.0 ± 3.9	**7.36 ± 0.69** **
B cells (%)	59.7 ± 0.8	**62.3 ± 0.5** *	62.6 ± 1.2	60.6 ± 0.5	60.9 ± 0.3	61.6 ± 1.0	**46.3 ± 0.5** **
NK cells	1.81 ± 0.14^##^	1.69 ± 0.13	1.41 ± 0.14	1.64 ± 0.14	**1.33 ± 0.11** *	**1.39 ± 0.14** *	**0.30 ± 0.03** **
NK cells (%)	2.51 ± 0.09^##^	2.51 ± 0.10	2.21 ± 0.19	2.49 ± 0.10	2.28 ± 0.14	**2.03 ± 0.08** **	**1.92 ± 0.06** **
Monocytes/ macrophages	1.57 ± 0.19	1.39 ± 0.11	1.48 ± 0.15	1.53 ± 0.10	1.32 ± 0.07	1.48 ± 0.18	**0.26 ± 0.03** **
Monocytes/ macrophages (%)	2.12 ± 0.09	2.04 ± 0.08	2.24 ± 0.14	2.29 ± 0.20	2.32 ± 0.15	2.11 ± 0.13	**1.64 ± 0.16** *
Neutrophils	0.76 ± 0.12	0.66 ± 0.07	0.91 ± 0.22	0.82 ± 0.22	0.53 ± 0.04	0.65 ± 0.08	**0.05 ± 0.01** **
Neutrophils (%)	1.00 ± 0.09	0.97 ± 0.06	1.40 ± 0.32	1.29 ± 0.40	1.01 ± 0.12	0.95 ± 0.09	**0.30 ± 0.03** **
Eosinophils	0.40 ± 0.07	0.28 ± 0.02	0.33 ± 0.05	0.31 ± 0.03	0.23 ± 0.02	0.28 ± 0.03	**0.06 ± 0.01** *
Eosinophils (%)	0.53 ± 0.06	0.42 ± 0.04	0.49 ± 0.07	0.46 ± 0.02	0.40 ± 0.03	0.42 ± 0.05	0.42 ± 0.07
Total T cell:B cell ratio	0.54 ± 0.02	0.48 ± 0.01	0.47 ± 0.03	0.50 ± 0.02	0.51 ± 0.01	0.51 ± 0.03	**1.05 ± 0.03** **
CD4^+^:CD8^+^ T cell ratio	1.67 ± 0.05	1.57 ± 0.03	1.64 ± 0.08	1.66 ± 0.04	1.63 ± 0.03	1.65 ± 0.05	**1.32 ± 0.05** **

Mean ± SEM (*N* = 7–8/treatment group).

Counts in 1 × 10^6^ cells/spleen.

CPS: cyclophosphamide (50 mg/kg); LUC: large unstained cell.

## Significant dose-related trend *p* < 0.01.

Bold values indicate significant pairwise differences when compared to the vehicle control group

* *p* < 0.05;

** *p* < 0.01.

**Table 6.** Splenic immunophenotypes following drinking water exposure to sulfolane in male and female F1 HSD rats.

	Sulfolane (mg/kg)					CPS (*N* = 8)
	0	30	100	300	1000	
F1 MALES						
Total spleen cells	510 ± 30.5^#^	509 ± 32.6	419 ± 23.0	511 ± 35.6	394 ± 37.9	**94.4 ± 9.8** **
Total Lymphocytes	482 ± 29.0	485 ± 31.3	398 ± 21.6	484 ± 33.9	373 ± 36.4	**91.4 ± 9.6** **
Total Lymphocytes (%)	94.6 ± 0.2	95.3 ± 0.3	95.0 ± 0.2	94.9 ± 0.2	94.6 ± 0.3	**96.8 ± 0.2** **
Total T cells	86.2 ± 5.6	92.6 ± 5.3	73.2 ± 4.0	93.4 ± 7.7	74.1 ± 8.4	**27.8 ± 3.2** **
Total T cells (%)	17.8 ± 0.5	19.2 ± 0.5	18.5 ± 0.4	19.2 ± 0.4	19.4 ± 0.5	**30.3 ± 0.7** **
CD4^+^ T cells	45.0 ± 2.5	49.3 ± 3.0	38.8 ± 2.2	49.1 ± 3.7	40.1 ± 4.7	**13.3 ± 1.4** **
CD4^+^ T cells (%)	52.7 ± 1.5	53.2 ± 1.3	53.2 ± 1.4	53.3 ± 1.8	54.2 ± 1.8	48.5 ± 2.3
CD8^+^ T cells	28.6 ± 2.7	32.0 ± 2.3	24.5 ± 1.6	32.8 ± 3.7	26.5 ± 3.4	**12.6 ± 1.7** **
CD8^+^ T cells (%)	32.8 ± 1.5	34.6 ± 1.5	33.6 ± 1.4	34.4 ± 1.6	35.6 ± 1.7	**44.9 ± 2.4** **
B cells	92.1 ± 9.3	90.7 ± 6.8	80.1 ± 5.7	98.1 ± 7.3	74.8 ± 8.6	**10.8 ± 1.2** **
B cells (%)	18.7 ± 0.7^#^	18.6 ± 0.5	20.0 ± 0.7	20.2 ± 0.7	19.8 ± 0.7	**11.7 ± 0.3** **
NK cells	44.6 ± 5.6^#^	42.1 ± 4.2	33.2 ± 2.4	37.8 ± 4.6	29.8 ± 3.5	**6.70 ± 0.73** **
NK cells (%)	8.99 ± 0.77	8.66 ± 0.63	8.42 ± 0.52	7.69 ± 0.62	8.11 ± 0.64	7.44 ± 0.63
Monocytes/ macrophages	15.1 ± 0.8^##^	15.1 ± 1.0	11.6 ± 0.7	15.5 ± 1.5	**10.4 ± 1.2** **	**1.80 ± 0.23** **
Monocytes/ macrophages (%)	3.02 ± 0.14	2.97 ± 0.07	2.78 ± 0.11	3.01 ± 0.15	2.67 ± 0.15	**1.87 ± 0.10** **
Neutrophils	13.9 ± 0.9^##^	13.2 ± 0.9	10.6 ± 0.8	12.4 ± 1.0	**9.39 ± 1.07** **	**4.59 ± 0.58** **
Neutrophils (%)	2.76 ± 0.14^#^	2.59 ± 0.07	2.51 ± 0.09	2.44 ± 0.15	2.37 ± 0.11	**4.87 ± 0.32** **
Eosinophils	1.90 ± 0.17	1.58 ± 0.12	1.38 ± 0.09	1.93 ± 0.17	1.37 ± 0.14	**0.64 ± 0.10** **
Eosinophils (%)	0.37 ± 0.02	0.32 ± 0.03	0.33 ± 0.02	0.38 ± 0.02	0.36 ± 0.02	**0.68 ± 0.08** **
Total T cell:B cell ratio	0.97 ± 0.05	1.04 ± 0.04	0.94 ± 0.04	0.96 ± 0.04	0.99 ± 0.04	**2.61 ± 0.11** **
CD4^+^:CD8^+^ T cell ratio	1.68 ± 0.15	1.59 ± 0.11	1.63 ± 0.10	1.62 ± 0.14	1.60 ± 0.14	**1.12 ± 0.12** *
F1 FEMALES						
Total spleen cells	364 ± 23.5	344 ± 19.4	308 ± 6.1	291 ± 15.1	384 ± 30.0	**65.5 ± 4.4** **
Total Lymphocytes	345 ± 23.0	329 ± 19.1	294 ± 5.7	276 ± 15.1	370 ± 29.3	**61.5 ± 4.2** **
Total Lymphocytes (%)	94.7 ± 0.9	95.5 ± 0.6	95.3 ± 0.4	94.8 ± 0.6	96.3 ± 0.4	93.9 ± 1.1
Total T cells	51.0 ± 3.0	50.4 ± 3.2	47.8 ± 1.4	43.7 ± 2.3	61.0 ± 5.2	**18.1 ± 1.2** **
Total T cells (%)	14.9 ± 0.4^##^	15.3 ± 0.4	**16.2 ± 0.3** *	**15.9 ± 0.3** *	**16.5 ± 0.5** *	**29.5 ± 0.9** **
CD4^+^ T cells	29.3 ± 2.2	29.3 ± 1.5	26.3 ± 0.8	24.6 ± 1.3	34.6 ± 3.5	**9.42 ± 0.61** **
CD4^+^ T cells (%)	57.0 ± 1.4	58.7 ± 1.4	55.1 ± 0.7	56.6 ± 1.6	56.2 ± 1.3	52.5 ± 2.2
CD8^+^ T cells	18.5 ± 1.0	17.8 ± 1.7	18.2 ± 0.9	15.9 ± 1.2	22.3 ± 1.8	**7.85 ± 0.77** **
CD8^+^ T cells (%)	36.6 ± 1.5	34.7 ± 1.4	37.9 ± 0.9	36.2 ± 1.4	36.9 ± 1.2	**43.0 ± 2.3** *
B cells	59.3 ± 4.9	55.8 ± 3.8	51.2 ± 2.0	48.6 ± 3.3	70.3 ± 7.1	**6.08 ± 0.66** **
B cells (%)	17.1 ± 0.6^#^	17.0 ± 0.7	17.4 ± 0.6	17.5 ± 0.6	18.8 ± 0.6	**9.81 ± 0.67** **
NK cells	40.7 ± 2.4	32.3 ± 2.7	34.4 ± 2.5	**30.6 ± 2.6** *	43.4 ± 4.5	**4.77 ± 0.63** **
NK cells (%)	12.1 ± 0.7	10.0 ± 0.7	11.7 ± 0.8	11.1 ± 0.8	11.8 ± 0.9	**7.61 ± 0.71** **
Monocytes/ macrophages	9.72 ± 0.72	9.21 ± 0.72	9.18 ± 0.36	8.78 ± 0.69	9.94 ± 1.24	**1.29 ± 0.14** **
Monocytes/ macrophages (%)	2.68 ± 0.13	2.67 ± 0.12	2.99 ± 0.13	3.02 ± 0.19	2.54 ± 0.13	**1.96 ± 0.12** **
Neutrophils	6.31 ± 0.50	5.62 ± 0.48	5.69 ± 0.35	5.66 ± 0.46	6.85 ± 0.64	**2.85 ± 0.46** **
Neutrophils (%)	1.76 ± 0.13	1.64 ± 0.10	1.85 ± 0.12	1.94 ± 0.09	1.79 ± 0.08	**4.37 ± 0.69** **
Eosinophils	1.17 ± 0.11	1.00 ± 0.07	0.95 ± 0.05	0.87 ± 0.07	1.15 ± 0.08	**0.46 ± 0.08** **
Eosinophils (%)	0.32 ± 0.02	0.29 ± 0.01	0.31 ± 0.01	0.30 ± 0.02	0.31 ± 0.02	**0.73 ± 0.12** **
Total T cell:B cell ratio	0.89 ± 0.05	0.92 ± 0.05	0.94 ± 0.04	0.92 ± 0.04	0.89 ± 0.04	**3.15 ± 0.29** **
CD4^+^:CD8^+^ T cell ratio	1.61 ± 0.11	1.74 ± 0.11	1.47 ± 0.05	1.61 ± 0.11	1.56 ± 0.09	1.27 ± 0.14

Mean ± SEM (*N* = 11–12/treatment group)

Counts in 1 × 10^6^ cells/spleen.

CPS, cyclophosphamide (15 mg/kg)

# Significant dose-related trend *p* < 0.05,

## *p* < 0.01.

Bold values indicate significant pairwise differences when compared to the vehicle control group

* *p* < 0.05;

** *p* < 0.01.
